# Highly Sensitive Multiplexed Detection of Brain‐Derived Tau and Phosphorylated Tau Proteoforms with the NULISA Technology

**DOI:** 10.1002/alz70856_101326

**Published:** 2025-12-25

**Authors:** Xiao‐Jun Ma, Shweta Iyengar, Shalaka Deshmukh, Roopa Comandor, Li Wang, Sean Kim, Xiaomei Xu, Wei Feng, Xiaolei Qiu, Bingqing Zhang, Yuling Luo

**Affiliations:** ^1^ Alamar Biosciences, Fremont, CA, USA

## Abstract

**Background:**

Blood‐based biomarkers hold great promise for the early detection and management of Alzheimer's Disease (AD). While plasma Aβ42/Aβ40 ratios and phosphorylated Tau forms (pTau‐181, pTau‐217 and pTau‐231) especially pTau‐217 have shown high diagnostic accuracies to identify abnormal Abeta pathology, brain‐derived Tau (BD‐Tau), a brain‐specific isoform of Tau, has demonstrated great promise as a blood‐based biomarker for distinguishing AD from non‐AD dementias and for predicting neurodegeneration. To address the need to measure BD‐Tau and other Tau forms in plasma in a single multiplex assay to evaluate their diagnostic performance and clinical utility, we sought to develop an ultrasensitive BD‐Tau assay with the novel Nucleic‐acid Linked Immuno‐Sandwich Assay (NULISA) technology. Incorporation of the BD‐Tau assay into the NULISAseq CNS Disease Panel 120 enables simultaneous profiling of multiple Tau proteoforms along with other important AD biomarkers in a single assay.

**Method:**

We developed a NULISA BD‐Tau assay by pairing a monoclonal antibody targeting MAPT exon 4‐5 junction with an antibody detecting total‐Tau (tTau). Detectability for BD‐Tau was evaluated in 100 plasma samples with the NULISA Singleplex assay. Integration of BD‐Tau into the CNS Disease Panel, a multiplex panel for detection of ∼120 key markers of neurodegeneration, inflammation and synaptic pathology, was assessed for interference with other Tau isoforms. Additionally, correlations were performed with t‐Tau assays in both plasma and CSF samples with singleplex and multiplex readouts.

**Result:**

The NULISA BD‐Tau assay provides highly specific detection of BD‐Tau with 100% quantifiability in both plasma and CSF (*n* = 86 each). As expected, BD‐Tau measurements showed very high correlation with t‐Tau in CSF samples (R=0.99), and lower correlation in plasma likely due to the presence of both peripheral and CNS‐derived Tau forms in blood. Addition of BD‐Tau to the CNS Disease Panel 120 showed minimal interference for detection of existing Tau forms (total Tau, t‐pTau‐181, t‐pTau‐217 and t‐pTau‐231) in plasma (*n* = 86).

**Conclusion:**

The NULISA BD‐Tau assay and its integration into the 120‐plex NULISAseq CNS Disease Panel provide powerful tools to develop blood‐based biomarkers for AD and enable deeper insights into AD pathogenesis and progression.

